# The role of ANGPTL3 in controlling lipoprotein metabolism

**DOI:** 10.1007/s12020-015-0838-9

**Published:** 2016-01-11

**Authors:** Anna Tikka, Matti Jauhiainen

**Affiliations:** National Institute for Health and Welfare. Genomics and Biomarkers Unit, Biomedicum, Haartmaninkatu 8, 00250 Helsinki, Finland

**Keywords:** Triglycerides, Lipoprotein metabolism, ANGPTL3, FHBL2, LPL

## Abstract

Angiopoietin-like protein 3 (ANGPTL3) is a secretory protein regulating plasma lipid levels via affecting lipoprotein lipase- and endothelial lipase-mediated hydrolysis of triglycerides and phospholipids. ANGPTL3-deficiency due to loss-of-function mutations in the *ANGPTL3* gene causes familial combined hypobetalipoproteinemia (FHBL2, OMIM # 605019), a phenotype characterized by low concentration of all major lipoprotein classes in circulation. ANGPTL3 is therefore a potential therapeutic target to treat combined hyperlipidemia, a major risk factor for atherosclerotic coronary heart disease. This review focuses on the mechanisms behind ANGPTL3-deficiency induced FHBL2.

## Introduction

Dietary fats are transported in the circulation in lipoprotein particles, lipid-apolipoprotein complexes containing a surface of phospholipid monolayer together with free cholesterol and structural apolipoproteins (apo) and a hydrophobic core including cholesterol esters and triglycerides (TG) [1, 2]. In humans, TGs are packed and secreted in the small intestine in apoB-48-containing chylomicrons (CM) and in the liver in apoB-100 containing very low density lipoproteins (VLDL) [1, 2]. TG in CM and VLDL are hydrolyzed in circulation by lipoprotein lipase (LPL) [3]. The resulting free fatty acids are taken up by tissues primarily via the function of CD36 transporter [4]. After deprivation of TG, CM, and VLDL remnants are cleared via specific liver receptors. Some VLDL remnants are converted in circulation, via hepatic lipase (HL) function, into cholesterol-rich low density lipoproteins (LDL). LDL-receptors recognize LDL-bound apoB-100, resulting in the uptake of the circulating LDL, mainly by the liver and by the steroidogenic tissues [3]. Another class of lipoproteins, high density lipoproteins (HDL), are functionally important in reverse cholesterol transport, to clear excess accumulated cholesterol from the periphery, and transport it back to the liver for excretion [5]. Disturbances in lipoprotein metabolism by genetic variants in genes affecting LPL activity (*ANGPTL3, APOC3, APOC2, APOA5*), remnant clearance (*APOE*, *LIPC*, *LRP1)*, LDL receptor activity (*PCSK9*, *LDLR*), lipoprotein secretion (*APOB, MTP*) and HDL (*APOA1, ABCA1*) have been detected in humans [6, 7].

High blood levels of saturated fat and cholesterol are major risk factors for coronary heart disease (CHD). Lipids within apoB-containing lipoproteins can accumulate in arterial intima and result in plaque formation and the development of atherosclerosis [8]. LDL-Cholesterol is a major cause for the generation of atherosclerotic plaques [8], however, an independent predictive value for elevated circulating TG in coronary heart disease (CHD) risk has been demonstrated in prospective studies [9, 10]. Existing pharmaceuticals, such as statins, fibrates, niacin, and fish oil, which target LDL-cholesterol (LDL-C), TG, and HDL-cholesterol (HDL-C) are prescribed alone or in various combinations to target dyslipidemias [11]. ANGPTL3 is a potential therapeutic target for alternative treatment of combined hyperlipidemia.

## Characteristics of ANGPTL3

The angiopoietin-like proteins (ANGPTLs) form collectively a specific family of secretory proteins sharing a structural similarity to angiopoietins, the key factors that regulate angiogenesis [12]. ANGPTL3 is a 460-amino-acid (aa) polypeptide with a distinctive signal peptide sequence, a N-terminal helical domain (predicted to form dimeric or trimeric coiled-coil structures) and a C-terminal globular fibrinogen homology domain [13]. The N-terminal coiled—coil region (17–207 aa), specifically the amino—acid domain 61–66, affects plasma triglyceride (TG) levels via reversibly inhibiting catalytic activity of LPL [14, 15]. The fibrinogen-like domain (207–460 aa) binds to integrin αvβ3 receptor and affects angiogenesis [16]. A short linker region (at 221–222 and 224–225) between N- and C-terminal domains functions as a furin cleavage site. The truncated form of cleaved ANGPTL3 displays enhanced inhibitory activity for LPL and endothelial lipase (EL) suggesting that furin-facilitated cleavage of ANGPTL3 is physiologically important [14, 17]. Angiopoietin-like protein 8 (ANGPTL8), an insulin-induced protein sharing sequence homology with ANGPTL3, may also regulate ANGPTL3 proteolytic activation in vivo [18–20].

The association of ANGPTL3 with lipoprotein metabolism was discovered in hypertriglyceridemic and hyperglycemic obese KK mice, which spontaneously inherited a recessive trait causing low levels of triglyceride in plasma [21] and elevated post-heparin LPL activity [22]. The hypolipidemic mice carried a 4-base pair insertion in exon 6 introducing a stop codon in the *Angptl3* gene. Low lipid levels in the mutant mice were normalized by an intravenous injection of ANGPTL3 [21]. When the region including *Angptl3* LOF was introduced to atherogenic apoE-knock out mice, the prevalence of atherosclerotic lesions significantly declined [23] indicating that ANGPTL3-deficiency and the resulting hypolipidemia in these mice, was protective against the development of atherosclerosis.

## Population studies and clinical characterization of *ANGPTL3* sequence variants in humans

In humans, both common and rare genetic variants in *ANGPTL3* gene have been reported to associate with plasma lipids. Three genome-wide scans (*n* = 8800) from Finnish and Italian subjects showed a strong association between *ANGPTL3* and plasma TG levels [6]. Since then, more data have been published on the association between *ANGPTL3* variants and plasma TG, LDL, HDL, and total cholesterol levels [24]. As much as 4 % of the Dallas Heart Study population (*n* = 3,551) with plasma TG in the lowest quartile carried rare LOF mutations in *ANGPTL3, ANGPTL4, or ANGPTL5* [25].

Genetic LOF variants in *ANGPTL3* cause a rare recessive disorder known as familial combined hypobetalipoproteinemia (FHBL2, OMIM # 605019). FHBL2 subjects display a distinct plasma lipid phenotype including very low VLDL, LDL, and HDL levels, and consequently low total TG and cholesterol levels ([26], see Table 1). Involvement of *ANGPTL3* LOF mutation in FHBL2 was discovered by genome sequencing of two siblings of European descent who were compound heterozygotes for two distinct nonsense mutations in *ANGPTL3* (p.E129* and p.S17*) [27]. p.S17* LOF mutation is common among the residents of a town Campodimele (Latina, Italy), of which 9.4 % carry the mutant variant [26]. Only the homozygous carriers of p.S17*, who have undetectable levels of ANGPTL3 in circulation, manifest low lipid and lipoprotein levels whereas heterozygote carriers, with a 50 % reduction in circulatory ANGPTL3, are not affected by FHBL2 (26, 34, see Table 1). In addition to p.S17* and p.E129* LOF mutations, more subjects with rare LOF variants in *ANGPTL3* are detected in Spanish and Italian FHBL2 families [28–30]. Among 78 sequenced American and Italian FHBL2 subjects, 8 subjects carried 9 different nonsense mutations in *ANGPTL3*, with no mutations in *APOB*, *PCSK9*, or *MTP*. The prevalence for *ANGPTL3* LOF mutations in all of the FHBL2 cases was therefore 10 % [31].

**Table 1:** Characteristic lipid, apolipoprotein, and Angptl3 levels of FHBL2 subjects with p.S17* mutation in the *ANGPTL3* gene

Parameter	Homozygote p.S17*-carriers (*n* = 5)	Heterozygote p.S17*-carriers (*n* = 17)	Non-carriers (*n* = 22)
Sex (W/M)	2/3	8/9	11/11
Age	63.6 ± 10	50.1 ± 20	51.8 ± 19
BMI	31.5 ± 7	28.3 ± 4	28.3 ± 5
Angptl3 (ng/mL)	0*	97 ± 96*	233 ± 145
FFA (µmol/L)	344.5 ± 292.2	486.2 ± 239.9	563 ± 249.9
ApoB (g/l)	1.0 ± 0.08*	1.5 ± 0.3	1.5 ± 0.4
ApoA-I (g/l)	0.5 ± 0.2*	1.3 ± 0.3	1.3 ± 0.2
TG (mmol/l)	0.5 ± 0.10*	1.1 ± 0.6	1.5 ± 0.7
CHOL (mmol/l)	2.3 ± 0.4*	4.6 ± 0.8	5.2 ± 1.1
LDL-CHOL (mmol/l)	1.4 ± 0.2*	2.6 ± 0.6	3.1 ± 0.8
HDL-CHOL (mmol/l)	0.7 ± 0.21*	1.5 ± 0.3	1.5 ± 0.4

Values in Table are modified from Robciuc et al. [34]. Values are reported as mean ± SD * *p* < 0.05. FFA values *N* = 7 for homozygotes, *N* = 47 for heterozygotes and *N* = 58 for non-carriers

## ANGPTL3 function

**Fig. 1:**
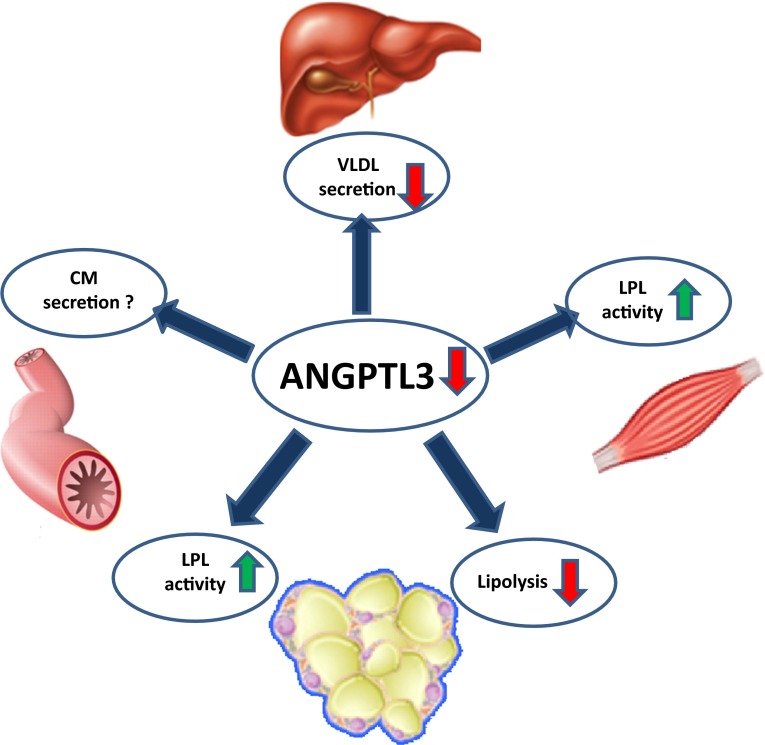
Function of ANGPTL3 in lipoprotein metabolism. ANGPTL3-deficiency causes enhanced activity of lipoprotein lipase (LPL) in the muscle and adipose tissue and accelerated clearance of TG-rich lipoproteins. Decreased release of FFA, from adipose tissue (lipolysis), hypothetically results in scarcity of FFA substrates for hepatic *de novo* synthesis of TG and cholesterol, and consequently decreased lipidation of VLDL. There are no reports on whether ANGPTL3-deficiency would affect the lipidation and secretion of intestinal TG-enriched chylomicron (CM)-particles

An increasing number of evidence indicates that the hypolipidemic phenotype in ANGPTL3-deficiency is driven by accelerated turnover of lipoproteins and the resulting altered energy substrate distribution among tissues (see Fig. 1).

### ANGPTL3 and lipoprotein clearance

Lipoprotein lipase (LPL) is located on the luminal side of the vascular endothelium of the capillaries in extra hepatic tissues [3]. LPL plays a critical role in hydrolyzing TG carried by VLDL and chylomicron (CM) particles in the circulation. Activity of LPL in white adipose tissue (WAT) is elevated in fed state and reduced in fasted state [3]. Therefore, in the fed state, the flow of dietary fat is primarily targeted into WAT, not into skeletal muscle, which would rely on glucose in energy production [3]. During fasting, skeletal muscle is believed to be the main site for LPL activity [3]. Endothelial lipase (EL) is located on the luminal side of the vascular endothelial cells (like LPL) and shares 44 % homology with LPL and 41 % with hepatic lipase (HL) [32]. Unlike LPL, EL is more specific in hydrolyzing lipoprotein phospholipids, especially in HDL-particles, rather than TG [33].

Both LPL and EL activities are increased in humans and mice with Angptl3 deficiency [34–36]. There are no reports on enhanced HL activity in ANGPTL3-deficiency. In *Angptl3*-knockout mice, TGs in postprandial lipoproteins were directed in brown adipose tissue (BAT) and muscle instead of WAT [37]. The study indicates that ANGPTL3-deficiency might induce LPL activity in the oxidative tissues during feeding and accelerate the clearance of TG-rich particles. Since LPL or EL do not hydrolyze cholesterol esters, the mechanism for low LDL-C levels in ANGPTL3-deficiency, has remained elusive. Wang et al. reported that LDL-cholesterol levels were equally reduced in wildtype mice and LDLR, LRP1, or APOE knockout mice treated with ANGPTL3 inactivating antibody [38]. These results suggest that reduced LDL-cholesterol in ANGPTL3-deficiency is not caused by enhanced clearance of cholesterol via LDL-receptors. Alternatively, reduced LDL-C levels may be a result of lower secretion rates of LDL precursors, hepatic VLDL particles.

ANGPTL3-deficiency in FHBL2 causes a reduction in HDL-particles, the facilitators of reverse cholesterol transport. As increased activity of EL is associated with reduced plasma HDL-cholesterol (HDL-C) levels [39], higher activity of EL might contribute to low HDL levels demonstrated in ANGPTL3-deficiency. Even though low HDL is an established risk factor for atherosclerosis [40], decreased HDL levels did not result in accelerated atherosclerosis in ANGPTL3-deficient subjects, probably because of the lifelong exposure to low levels of VLDL and LDL [30]. The lipolysis of TG-rich lipoproteins and HDL, via LPL and EL, respectively, may result in increased turnover of lipoprotein remnants and therefore low plasma apoB and apoA-I levels.

### The role of ANGPTL3 in lipoprotein production

Hepatic VLDL synthesis relies on the availability of triglycerides which are synthesized from substrates derived from the supply of free fatty acids from adipocytes [41], from VLDL and chylomicron remnants [1, 2] and from simple sugars [42] via the portal vein. Insulin signaling reduces hepatic VLDL synthesis and secretion which is manifested by reduced lipidation of TG-rich VLDL particles [43]. Mice treated with monoclonal ANGPTL3 inactivating antibody did not show lower production rates for apoB-100 [38], the major structural protein of VLDL, indicating that there are no changes within the amount of secreted VLDL particles. However, the amount of TG in each VLDL particle might be declined [44]. Reduced lipidation of VLDL in ANGPTL3-deficiency may be caused by decreased supply of free fatty acids from the circulation into the liver. These observations are in agreement with the reported low FFA levels in ANGPTL3-deficient subjects [34]. A metabolic study with ANGPTL3-silenced hepatocytes suggests that silencing of ANGPTL3 causes a shift in substrate utilization to favor glucose, instead of FFA [44], which supports the theory of attenuated FFA supply into the liver.

Cholesterol is either ingested from nutrition or synthesized *de novo* by the liver. Reduced availability of substrates may decrease hepatic *de novo* synthesis of cholesterol resulting in the secretion of cholesterol-poor VLDL in ANGPTL3-deficiency. Interestingly, *ANGPTL3* is a downstream target for liver X receptors (LXR’s), transcription factors with sterol binding ability [45]. Oxysterols, cholesterol, and other cholesterol metabolites are natural ligands for LXRs, and LXR activation protects the liver from cholesterol overload by stimulating bile acid formation and excretion, lipogenesis, HDL-mediated reverse cholesterol transport, and glucose metabolism [46, 47]. Synthetic LXR ligands and a high cholesterol diet induce *Angptl3* expression in the liver [48, 49]. On the contrary, low LXR, and low *Angptl3* expression (in ANGPTL3-deficiency) may therefore be linked to hypocholesterolemic and antilipogenic status in the liver.

### Adipose tissue

White adipose tissue (WAT) serves as the primary tissue for storage of triglycerides. Postprandial TGs are primarily directed to WAT via LPL-mediated hydrolysis [3]. In *Angptl3*-knockout mice, TG-derived FFA uptake was decreased in WAT while elevated in BAT and muscle. Such a shift in substrate distribution between tissues, however, did not result in differences in TG-content of the liver, adipose tissue, and heart, possibly because reduced lipid uptake in WAT was balanced by elevated glucose uptake [37].

TG-deposits in adipose tissue are dismantled by lipolysis, a catabolic breakdown of TG mediated by concerted action of adipose TG lipase (ATGL), and hormone sensitive lipase (HSL) [41]. Lipolysis is inhibited in the postprandial state by elevated insulin concentrations [50]. During fasting, lipolytic activity increases the release of FFA and free glycerol into circulation, to provide substrates for energy production in the oxidative tissues [41]. In *Angptl3*-knockout mice, both FFA and glycerol were reduced in plasma and were preserved by administration of ANGPTL3 [51]. Therefore, absence of circulatory ANGPTL3 (in ANGPTL3-deficiency) may decrease FFA lipolysis in the adipose tissue and consequently reduce plasma FFA and glycerol levels.

## Conclusions

Several mutations in the *APOB*, *PCSK9*, and *MTP* genes result in familial hypobetalipoproteinemia (FHBL) and abetalipoproteinemia (ABL) defined as low, or absent levels of apoB-100, and LDL-C in plasma [52, 53]. LOF mutations in the *ANGPTL3* results in familial combined hypobetalipoproteinemia (FHBL2) with reduced levels of all major lipoprotein classes (VLDL, LDL, HDL) in plasma. The hypolipidemic phenotype caused by ANGPTL3 inactivation is likely an outcome of increased activity of two lipolytic enzymes, LPL, and EL, which account for increased turnover of lipoproteins, and reduced TG and HDL levels in the plasma. Low plasma FFA levels in ANGPTL3-deficiency may be due to decreased lipolysis in the adipose tissue which may also contribute to reduced lipidation of hepatic VLDL, and LDL, and consequently low TG and LDL-C. The impact of ANGPTL3-deficiency on intestinal chylomicron production remains unknown. However, there are no reports on steatorrhea among ANGPTL3-deficient subjects.

No adverse health effects or developmental alterations during early or later life-time are reported in ANGPTL3-deficient p.s17* LOF mutation carriers [26, 34]. Perhaps ANGPTL3 may have been functionally important during long periods of fasting to primarily ensure FFA release from TG-deposits, via controlling lipolytic activity, and by guiding energy substrates into either oxidative tissues or WAT by selective LPL inhibition. Such tight regulation between energy storage and substrate release might be important during times with less frequent access to energy rich diet.

The striking hypolipidemic phenotype in ANGPTL3 deficiency shows the potential of ANGPTL3 inactivation as a treatment for correcting hyperlipidemia. Therefore, ANGPTL3 inactivation may have important therapeutic implications for treatment of metabolic syndrome, type 2 diabetes, and patients in high risk of heart disease. The first reports on ANGPTL3 inhibitors have shown promising results. Administration of ANGPTL3 inactivating antibody to monkeys and dyslipidemic mice reduced circulating plasma levels of TG, LDL-cholesterol, and HDL-cholesterol significantly [54]. Another strategy for ANGPTL3 inactivation, and perhaps a better one to avoid immune response associated with administration of antibodies, could be selective antisense inhibition of *ANGPTL3* expression in the liver. Despite favorable reduction in LDL-C and TG, it is still uncertain whether decreased HDL-C would have an influence on overall CVD risk in ANGPTL3-inhibitor treated patients.
